# Transverse Fracture of the Axis

**Published:** 1898-02

**Authors:** G. L. Warner

**Affiliations:** New York City


### REPORTS OF CASES.

### TRANSVERSE FRACTURE OF THE AXIS.

By G. L. Warner, D.V.S.,
NEW YORK CITY.

*Subject.* A black gelding, twelve years old; blind.

*History.* Following a broken bit, the animal ran away with a loaded truck, for about one hundred feet, and came in contact with a brick wall, with his head, with such force as to throw the animal down, remaining prostrate and still for about three minutes, when he gradually recovered consciousness and arose without assistance. After blanketing and resting for a short time, he seemed sufficiently recovered to resume his work, delivering the load upon the truck, and returning a distance of three miles to his owner's place of business. The only difference noticed by the driver was that the animal did not pull as hard on the bit as usual, and apparently staggered at times; and about three hours after the accident a swelling commenced to show itself on the side of the neck over the axis. The accident occurred at 8 A.M., and the animal continued in service until 5.30 P.M. I visited him at 6.30 P.M., and noted the following symptoms:

*Physical Examination.* The head turned considerably to the right; animal staggered when walking; a large swelling over the axis on the left side. Temperature normal; eating hay. Any attempt to turn the head to the left induced pain and uneasiness.

*Diagnosis.* Fracture of the axis, and advised destruction of the animal. This was deferred, and I revisited him the following morning and found the swelling increased, the head still further turned to the right; had not lain down; eating fairly well and temperature 102° F.; increased unsteadiness in walking, and disinclined to move, holding back on the halter. Owner consented to have him destroyed.

*Autopsy,* held twenty-four hours later. Subcutaneous tissues stained and filled with serosity, the muscular structures bruised, with clots of blood; the axis fractured transversely posterior to the odontoid process; fracture simple, with the exception of a few small fragments; the dura mater stripped from the spinous canal for about one inch anteriorly and posteriorly to the break; the membranes were intact, but an exudation of blood between them and the cord, which was stained upon its surface with blood.

The extent of the fracture and lesions found were such as to cause much surprise at the mild character of symptoms exhibited during life, taken in conjunction with the work performed after the accident and to having ascended two runs, fifty feet long, to the third floor of the stable where kept, living forty-eight hours with a broken neck.

